# Two case reports of corticosteroid administration-prolonged and pulsed therapy-in treatment of pruritus in cholestatic hepatitis A patients

**DOI:** 10.1093/omcr/omz080

**Published:** 2019-08-26

**Authors:** Daad Daghman, Mohamad Saeed Rez, Amjad Soltany, Almotaman Alsaleh

**Affiliations:** 1 Teacher of Gastroenterology, Department of Gastro-enterology Faculty of Medicine, Tishreen University, Lattakia, Syria, Teacher of Gastroenterology at Al Andalus University for Medical Science, Tartous, Syria; 2 Department of Neurology, Al-Bassel Hospital, Tartus, Syria; 3 Department of Plastic and Reconstructive Surgery, Al Mouwasat University Hospital, Damascus, Syrian Arab Republic; 4 Department of Cardiology, Tishreen University Hospital, Lattakia, Syria

## Abstract

Cholestasis following hepatitis A affects around 0.8% of hepatitis A patients. It is considered a distressing complication in spite of its good prognosis. Despite being subject to multiple studies, causes of cholestasis are still controversial. Many treatments (discussed later) have shown some improvements of the accompanied pruritus. In the following article, we present two cholestatic hepatitis A patients who suffered from severe pruritus. Prednisolone was administered via two different methods: prolonged and pulsed. Both showed great improvement of the pruritus in a short time frame. To the best of our knowledge, our management using pulsed corticosteroid therapy in treatment of pruritus in cholestatic hepatitis A is considered the first experimental management in medical literature. The importance of this experimental case lies in reducing the doses and the duration of steroid intake, thus reducing steroid side effects as far as possible.

## INTRODUCTION

Hepatitis A is considered a self-limiting infectious disease with neither specific treatment nor recommended diet. Its rare complications include recurrence, acute liver failure and cholestasis. Cholestatic hepatitis is accompanied by elevated liver enzymes and increases in blood bilirubin levels, in addition to pruritus [1]. As this pruritus may be a gravely disabling symptom, numerous evidence-based therapeutic strategies (discussed later) are recommended.

## CASE REPORT

### Case 1

A 21-year-old man presented with seriously disabling pruritus, nausea and vomiting, in addition to general fatigue 10 days prior to the pruritus. He was afebrile on admission, and physical examination revealed jaundice with mild hepatomegaly. Admission work-up is shown in Table 1.

**Table 1:** 

Bilirubin Normal Range: 0-1 mg/dl	Alanine Aminotransferase (ALT) Normal Range: 5-45 U/l	Aspartate Aminotransferase (AST) Normal Range: 5-40 U/l	Antibodies against hepatitis C virus (Anti HCV)	Hepatitis B surface antigen (HBsAg)
10.9 mg/dl	199 U/l	270 U/l	Negative	Negative
anti-smooth muscle antibody (ASMA)	Anti-mitochondrial antibodies (AMA)	Antinuclear Antibodies (ANA)	HAV IgM antibody( anti-HAV IgM) Positive: more than 1.0	Ceruloplasmin Normal Range: 20–35 mg/dl.
Negative	Negative	Negative	1.6 (positive)	25, 4mg/dl.

**Table 2:** 

Bilirubin	Direct Bilirubin normal range: 0-0.25 mg/dl	Alanine aminotransferase (ALT)	Aspartate aminotransferase (AST)
22.5 mg/dL	20.94 mg/dL	32 U/l	45 U/l
Gamma-glutamyl transferase (GGT) Normal Range: 9–39 U/l	Prothrombin time	International normalized ratio (INR)	Alkaline phosphatase (ALP) Normal range: 64–306 U/l
22 U/l	58%	1.34	390 U/l

**Table 3:** 

Bilirubin	Direct bilirubin	Alt	AST	PT
15.9 mg/dl	14.3 mg/dl	30 U/l	61 U/l	100%

**Table 4:** 

Bilirubin	Direct Bilirubin	PT	INR
7.8 mg/dl	6.9 mg/dl	100%	1

An abdominal ultrasound showed mild hepatosplenomegaly. The patient was diagnosed with hepatitis A (on the basis of elevated hepatitis A virus (HAV) IgM antibodies and negative serology for hepatitis (B, C) accompanied with cholestasis as a following complication. No specific treatment or diet was recommended expect for the pruritus which was firstly treated by ursodeoxycholic acid and antihistamines, but there was no improvement. Four weeks later, follow-up laboratory values are shown in Table 2.

The pruritus continued causing notable weight loss and insomnia. Several bruises and petechiae appeared on the skin of arms and thighs. Then, treatment with prednisolone started with an initial dose of 40 mg, tapered weekly by 5 mg. Four days later, the pruritus started to reduce and the bilirubin values also decreased (Table 3).

**Figure 1:**
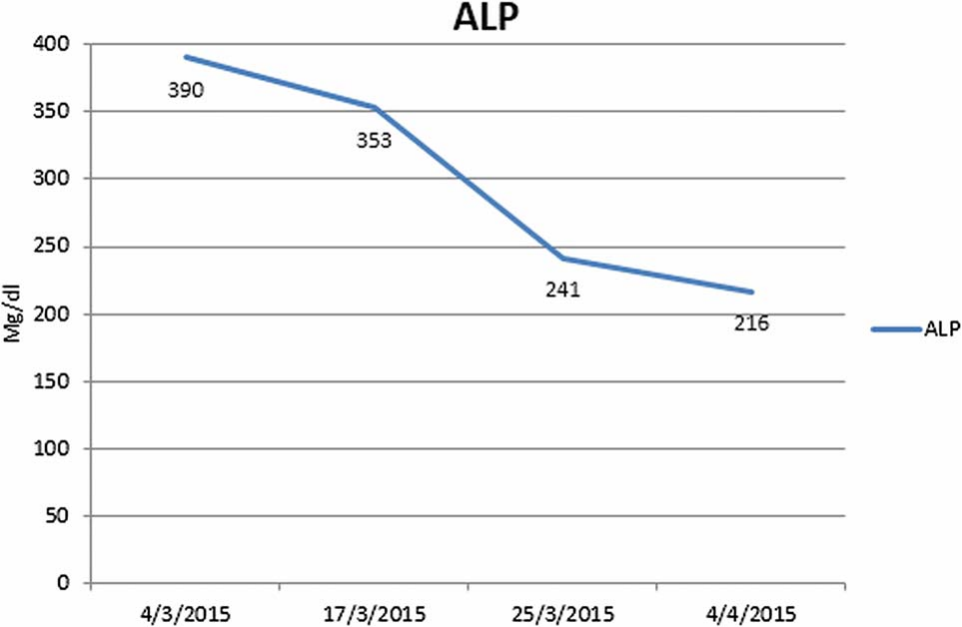
Blood alkaline phosphatase (ALP) levels in patient 1.

**Table 5:** 

Bilirubin	Direct Bilirubin	ALT	AST	GGt	ALP Normal Range: 64-306 U\l
1.4 mg/dl	1 mg/dl	1440 U/l	1500 U/l	168 U/l	110 U/l
HAV IgM Positive: more than 1.0	HBsAg	Anti HCV	Ceruplasmine	ANA	ASMA
2.94 (positive)	Negative	Negative	24 mg/dl	Negative	Negative

**Table 6:** 

Bilirubin	Direct bilirubin	ALT	AST
12.9mg/dl	10 mg/dl	1705 U/l	545 U/l
ALP	PT	INR	
180 U/l	59%	1.46	

**Table 7:** 

Bilirubin	Direct bilirubin	ALT	AST	Alkaline phosphatase (ALP)	GGT
10.38 mg/dl	8.9 mg/dl	380 U/l	55 Ul/	230 U/l	100 U\l

**Table 8:** 

Bilirubin	Direct bilirubin	ALT	AST
3.3mg/dl	2.5 mg/dl	60 U/l	55 U/l
GGT	ALP		
101 U\l	125 U\l		

Six days later, bilirubin values continued to decrease (Table 4).

Following 4 weeks of treatment with prednisolone, total bilirubin level reached 2.27 mg/dl. See Fig. 1.

### Case 2

A 30-year-old female presented with nausea, vomiting and anorexia. Physical examination revealed tenderness in the right hypochondrium. No other significant clinical findings in her physical examination were found.

Admission work-up is shown in Table 5.

The patient was diagnosed with hepatitis A (on the basis of elevated HAV IgM antibodies and negative serology for hepatitis B, C). Five days later, jaundice and pruritus became apparent.

The anorexia persisted for a week accompanied with severe nausea and pruritus.

Follow-up laboratory values are shown in Table 6. Ten days later, the severe pruritus persisted and laboratory values were repeated again (Table 7).

**Figure 2:**
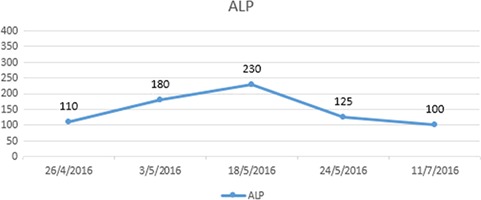
Blood alkaline phosphatase (ALP) levels in patient 2.

On the same day, prednisolone was administered as a pulsed therapy for 4 days starting with 30 mg for the first day, then 20 mg, 10 mg and 10 mg once daily, respectively, for each of the following days. The pruritus gradually improved on the second day and disappeared completely at the end of the fourth day with values shown in Table 8. Five weeks later, the follow-up laboratory values are shown in Table 9. See Fig. 2.

## DISCUSSION

Hepatitis A is a self-limiting disease where recovery usually starts at around 7–14 days following the onset of symptoms. Hepatitis A can become cholestatic in about 0.8% of patients [2]. Cholestasis is a result of impaired metabolism and flow of bile in the liver [3]. Many different causes of this issue have been proposed. These causes include genotypes and sub-types of hepatitis A viruses. It was discovered that the sub-types Ia and Ib were responsible for the long-term nature of the disease because of the difficulty of producing antibodies against these two sub-types [1]. The exchange of nucleotides in the mid-section of the untranslated sector 5 may also intervene with the severity of the damage [1]. In addition, cytokines and other inflammatory promoters like TNF-α and IL1 play a role in causing cholestasis [2, 4].

The virus may also cause spotty necrosis in the periportal space that interrupts the continuity of bile flow to portal ducts due to secondary immune reaction [5]. Cholestatic hepatitis also causes interlobular bilirubin accumulation especially in zone 3 as well as necrosis in the infected hepatocyte caused by cluster of differentiation 8 (CD8) positive T cells [6], in addition to endotoxin released by the liver [2].

**Table 9:** 

Bilirubin	Direct bilirubin	ALT	AST	GGT	ALP
0.75 mg/dl	0.5 mg/dl	24 U/l	35 U/l	16 U/l	100 U/l

**Table 10:** 

Bile salts	Bile salts play a major role in pruritus, but several studies observed that there is no connection between the severity of the pruritus and the concentration of different bile salts in plasma, although it has been confirmed that bile salts have an indirect effect that needs further studies [7].
Lysophosphatidic acid (LPA)	Lysophosphatidic acid (LPA) also plays a role in pruritus since its concentration increases in patients with cholestasis, this was explained as a result of over-expressing autotaxin gene, leading to over-converting lysophosphatidyl choline (LPC) to lysophosphatidic acid [8].
Opiates	Opiates were also found to play a role in pruritus by activating μ receptors which may cause pruritus.[3–8].
Progesterone sulfates	Progesterone sulfates and other products of cholestasis could alter the neurological reaction on both spinal cord and brain levels [3–8].
Other compounds	Serotonin, gastrin-releasing peptide (endovanilloid) and their receptors. - Transient receptor potential cation channel sub-family V member 1(Tprv-r), capsicin-r as well as cannabis, gamma-aminobutyric acid (GABA) and histamine inhibitors have been mentioned [3–8].

**Table 11:** 

Date	Bilirubin	Date	Bilirubin
4/3/2015	10.9 mg/dl	4/4/2016	1.4 mg/dl
17/3/2015	16.3 mg/dl	3/5/2016	10.4 mg/dl
25/3/2015	19.5 mg/dl	18/5/2016	12.9 mg/dl
28/3/2015	22.5 mg/dl		
30/3/2015	Corticosteroids Therapy Started	19/5/2016	Corticosteroids Therapy Started
4/4/2015	15.9 mg/dl	24/5/2016	3.3 mg/dl
9/4/2015	11.8 mg/dl	11/7/2016	0.8 mg/dl
15/4/2015	7.8 mg/dl		
3/5/2015	2.27 mg/dl		

The compounds that are thought to have a role in triggering pruritus in patients with cholestasis following hepatitis A are shown in Table 10.

As a result of all these studies, many treatments were found for pruritus which is considered the most distressing and persistent symptom in patients with cholestatic complications. However, no final treatment is recommended since drugs react differently in different patients.

One of these treatments is ursodeoxycholic acid (UCDA) that lowers levels of transient bile salts and levels of bilateral sulfide steroids hence reducing the pruritus [7]. Additionally, there is a group of drugs that work on promoting metabolism of liver by stimulating cytochrome P 450 enzymes, such as rifampicin and phenobarbital [3].

Opioid inhibitors such as naltrexone could be used in addition to anesthetic drugs like lidocaine, antihistamines and resins that prevent the reabsorption of bile salts from the intestines such as cholestyramine. Serotonin reuptake inhibitors such as sertraline, phototherapy and molecular adsorbent recirculating system (MARS) are also administrated for this purpose [5]. Corticosteroids have shown considerable efficacy in achieving symptom resolution in previous studies [8–9].

As for our previous two patients, they were given prednisolone in two different ways; the male was given steroids for 10 weeks with a starting dose of 40 mg tapered by 5 mg weekly (conventional or prolonged therapy). The female was given 30 mg for the first day, then 20 mg, 10 mg and 10 mg, respectively, for each of the following 3 days (pulsed therapy). Both patients showed marked improvement and the distressing pruritus decreased after a few days of therapy (Table 11). It is notable that the male showed a better prothrombin time directly after being given prednisolone.

It is thought that the mechanism of action of corticosteroids depends on its anti-inflammatory effects and improving the mpr2 protein’s actions that increases the transport rates of bilirubin to outside of the hepatocytes making it more efficient [9].

Administration of corticosteroids may improve the prognosis of cholestatic hepatitis A as well as relieve the accompanied pruritus, so we recommend physicians to publish the same cases to be able to determine the accurate criteria for pulsed versus prolonged steroid therapy. Eventually, we still emphasize that corticosteroid pulsed therapy could be clinically effective thus reducing corticosteroid administration time, decreasing its considerable side effects.

## Funding

None to declare.

## The Guarantor

Amjad Soltany, MD.

## Consent

Written consent received.
